# Meeting Report From the 2024 Symposium on IRAK4 in Cancer: Highlights and Clinical Updates

**DOI:** 10.1016/j.clml.2025.05.020

**Published:** 2025-05-30

**Authors:** Eric S. Winer, Marina Konopleva, Han W. Tun, Kian-Huat Lim, Bently Doonan, Klaus H. Metzeler, Lakshmi Nayak, Andrés J.M. Ferreri, Christina von Roemeling, Grzegorz S. Nowakowski, Guillermo Garcia-Manero

**Affiliations:** 1 Department of Medical Oncology, Dana-Farber Cancer Institute, Boston, MA; 2 Department of Oncology, Albert Einstein College of Medicine, Bronx, NY; 3 Department of Molecular Pharmacology, Montefiore Einstein Comprehensive Cancer Center, Bronx, NY; 4 Department of Hematology, Mayo Clinic Florida, Jacksonville, FL; 5 Department of Medicine, Washington University School of Medicine in St. Louis, St. Louis, MO; 6 Division of Hematology and Oncology, University of Florida College of Medicine, Gainesville, FL; 7 Hematology, Cell Therapy and Hemostaseology, University of Leipzig Medical Center, Leipzig, Germany; 8 Center for CNS Lymphoma, Dana-Farber Cancer Institute, Boston, MA; 9 Lymphoma Unit, IRCCS San Raffaele Scientific Institute, Milan, Italy; 10 Department of Oncology, Università Vita-Salute San Raffaele, Milan, Italy; 11 Department of Neurosurgery, University of Florida College of Medicine, Gainesville, FL; 12 Division of Hematology, Mayo Clinic Comprehensive Cancer Center, Rochester, MN; 13 Department of Leukemia, University of Texas MD Anderson Cancer Center, Houston, TX

**Keywords:** AML, CA-4948, Emavusertib, MDS, PCNSL

## Abstract

Interleukin-1 receptor-associated kinase 4 (IRAK4) is a serine/threonine kinase that mediates interleukin-1 and Toll-like receptor (TLR) signaling. IRAK4 drives the activation of nuclear factor kappa B (NF- *κ*B), which promotes cell survival, inflammation, and proliferation. Aberrant activation of IRAK4 and TLR signaling has been implicated in multiple malignancies. At the 3rd Annual IRAK4 in Cancer Symposium, experts discussed the role of IRAK4 in cancer biology, the potential for synergism between IRAK4 inhibition and other treatments to overcome resistance, and how IRAK4 inhibition may improve clinical outcomes. Preclinical data were presented demonstrating the activity of IRAK4 inhibition alone or in combination with other anticancer agents in acute myeloid leukemia, myelodysplastic syndrome, non-Hodgkin lymphoma, primary central nervous system lymphoma, melanoma brain metastases, gastrointestinal cancers, and pancreatic ductal adenocarcinoma. Clinical data from the targeted small-molecule IRAK4 inhibitor emavusertib (CA-4948) were presented, including data from the TakeAim Leukemia and TakeAim Lymphoma trials of emavusertib in myeloid and lymphoid malignancies, respectively, and preliminary data from trials of emavusertib in multiple solid tumors. The meeting closed with expert discussion of the emerging profile of IRAK4 inhibition in cancers and the potential for IRAK4 inhibition to improve outcomes across both solid and liquid tumors.

## Introduction

### Overview of IRAK4 in Cancer

**Figure 1:**
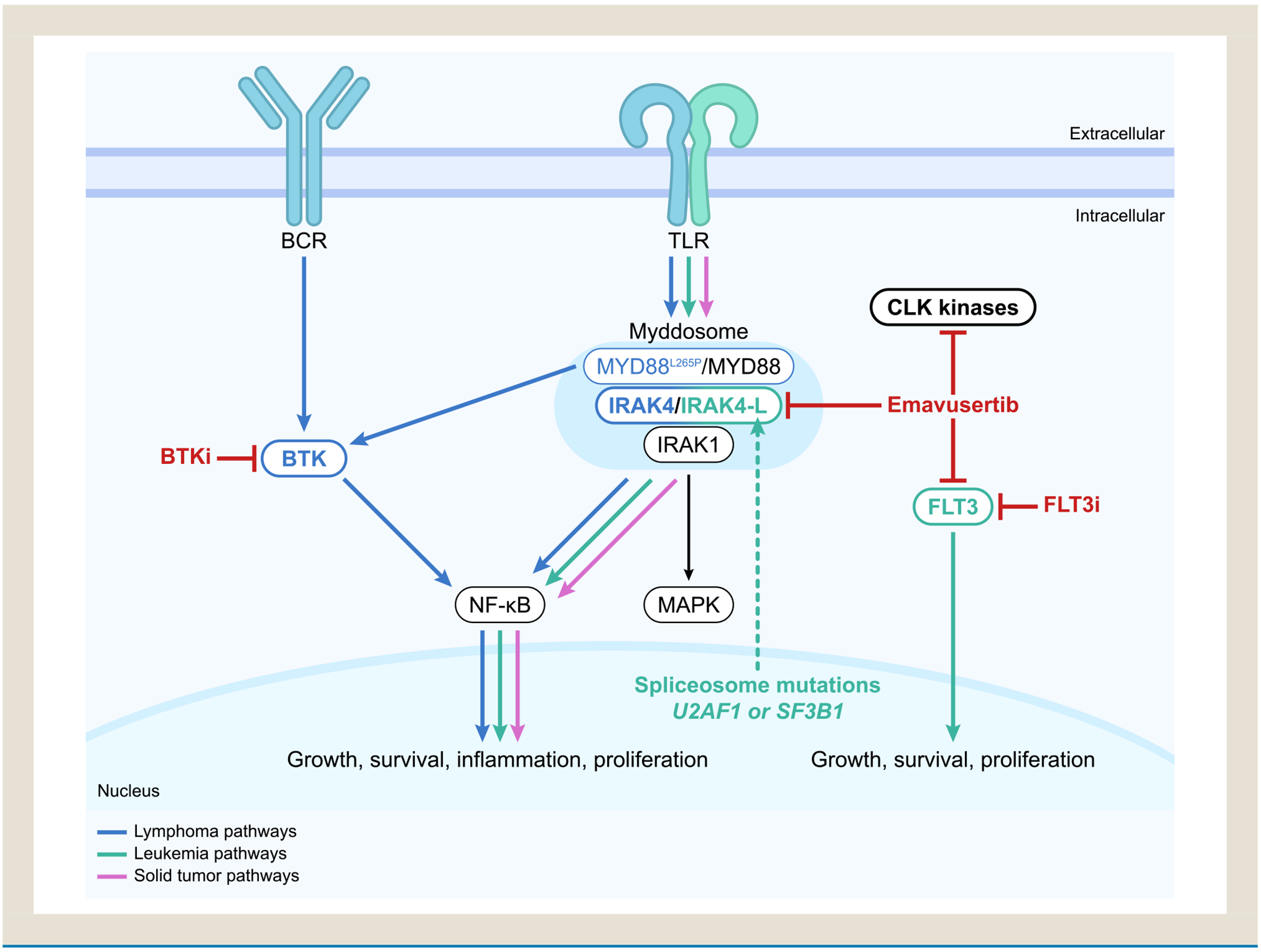
IRAK4 signaling in malignant pathways and approach to inhibition. Key BCR, TLR, and IRAK4 signaling pathways discussed during the symposium. Both emavusertib and R835 inhibit TLR signaling via inhibition of IRAK4 and IRAK1/4, respectively.^2, 12, 13^ In AML, emavusertib targets TLRs, *FLT3*, DYRK1A, and CLKs, all of which have downstream leukemic effects.^2, 3, 10, 14, 18–21^ Based on preclinical data, there is potential for emavusertib to overcome resistance to FLT3 inhibitors and to BCL2 inhibitors, especially if the latter is due to activating mutations in *FLT3* or the RAS/MAPK pathway or increased MCL-1 expression.^21, 25–27^Additionally, in patients with AML, emavusertib treatment reduced the variant allele frequency of *FLT3m*, in some cases to below the limit of detection.^38^ Emavusertib has been found to potentiate the antitumor effects of azacitidine and venetoclax in azacitidine- and venetoclax-resistant AML cell lines, and thus the combination of azacitidine, venetoclax, and emavusertib has the potential to deepen responses.^28^ In NHL models, combinations of emavusertib and BTK inhibitors such as ibrutinib, zanubrutinib, or acalabrutinib have shown synergistic effects via inhibition of both TLR and BCR signaling.^17, 40–42^ Emavusertib plus ibrutinib has shown encouraging outcomes in patients with PCNSL. In select solid tumors such as MBM, PDAC, and gastrointestinal cancers that generally have limited responses to immunotherapy, activation of IRAK4/TLR signaling contributes to proinflammatory immune microenvironments.^42, 45, 46, 48^ IRAK4 inhibition has the potential to modulate these environments, and addition of emavusertib to immune checkpoint inhibitors has shown increased antitumor activity in models, likely via interruption of the inflammatory cascade.^17, 45, 46^ Addition of emavusertib to chemotherapy has also resulted in increased antitumor responses in PDAC and CRC models.^45, 46^ Figure adapted from Garcia Manero, et al. *Front Hematol*. 2024;3:1339870.^2^ Abbreviations: BCR = B-cell receptor; BTK = Bruton’s tyrosine kinase; CLK = CDC-like kinase; FLT3 = FMS-like tyrosine kinase 3; i = inhibitor; IRAK1 = interleukin 1 receptor-associated kinase 1; IRAK4 = interleukin 1 receptor-associated kinase 4; MAPK = mitogen-activated protein kinase; MyD88 = myeloid differentiation primary response; NF-B = nuclear factor kappa B; TLR = toll-like receptor.

Interleukin-1 receptor-associated kinase 4 (IRAK4) is a serine/threonine kinase that mediates interleukin 1 and Toll-like receptor (TLR) signaling as a core component of the myddosome (Figure 1).^1, 2^ The myddosome—comprising IRAK4, IRAK1/2, and myeloid differentiation primary response 88 (MYD88)—assembles in response to receptor engagement and results in nuclear factor kappa B (NF- *κ*B) and mitogen-activated protein kinase (MAPK) activation.^1, 2^ NF- *κ*B and MAPK pathway signaling is associated with activation of the innate immune system, increased cell survival, and proinflammatory cytokine production.^2–4^ Spliceosome mutations that occur in both acute myeloid leukemia (AML) and myelodysplastic syndrome (MDS) result in preferential splicing of the *IRAK4* mRNA to a long isoform (IRAK4-L) that results in maximal activation of NF- *κ* B.^5–9^ Dysregulation of TLR signaling contributes to the pathogenesis of many hematologic malignancies and solid tumors, making IRAK4 a promising target for anticancer therapeutics.^2^ IRAK4 pathway biology and its role in malignancies have been discussed in detail in recent reviews by Garcia-Manero, et al., and Parrondo, et al.^2, 10^

### Targeting IRAK4

There is significant clinical interest in targeting IRAK4 and, as of the time of this report, there are 2 IRAK4-targeted agents under clinical investigation in oncology: R835/R289 (Rigel Pharmaceuticals, Inc., San Francisco, CA, USA) and emavusertib (CA-4948; Curis, Inc., Lexington, MA, USA).^10, 11^

R835 is a selective dual inhibitor of IRAK1/4 that blocks TLR4 and interleukin 1 receptor-dependent cytokine release and has been studied preclinically in rheumatological disease. Initial studies of R835 demonstrated a blockade of TLR4/lipopolysaccharide (LPS) ligand signaling and the downstream production of pro-inflammatory cytokines in both human and mouse cells.^12^ Furthermore, in a randomized, dose-escalation, placebo-controlled, double blind Phase 1 study in healthy adults, this drug was found to be well tolerated and demonstrated inhibition of the acute release of TNF- *α*, IL-6, IL-8, MIP1*α*, and MIP1*β*.^13^

R289 is a pro-drug that is converted to R835 in the gut and is under investigation in MDS. R289 is currently being assessed in a Phase 1b study of patients with lower-risk MDS who are refractory/resistant to prior therapies (NCT05308264).^11^ As of July 15, 2024, the most frequently reported treatment-emergent adverse events (TEAEs) were diarrhea and fatigue (5/19 patients, 26% for each); the most frequent Grade 3-4 TEAEs were pneumonia, anemia, increased alanine aminotransferase, and upper gastrointestinal hemorrhage (2/19 patients, 10% for each). Of 14 evaluable patients, 3 achieved red-blood cell transfusion independence (RBC-TI) > 8 weeks (22%) and 2 achieved RBC-TI > 24 weeks (14%).^11^

Emavusertib is a first-in-class, orally available, selective small-molecule inhibitor that binds to the IRAK4 kinase domain and blocks the IRAK4-dependent phosphorylation of IRAK1 and thus downstream activation of MAPK and NF- *κ*B.^14, 15^ Emavusertib exhibits rapid absorption, stability in plasma, a 6-hour half-life, and the ability to cross the blood–brain barrier.^16, 17^ Emavusertib also targets FMS-like tyrosine kinase 3 (FLT3)—mutated forms of which are common driver mutations and components of resistance mechanisms in AML-and the spliceosome factor regulators CDC-like kinases (CLKs) and dual-specificity tyrosine-regulated kinase 1 (DYRK1), which regulate the splicing of key pro-survival factors critical in oncogenesis.^14, 18–21^ Activation of innate immune signaling through IRAK4 contributes to the development of resistance to FLT3 inhibitors;^22^ dual inhibition of FLT3 and IRAK4 has the potential to ameliorate this resistance pathway.

Emavusertib is currently under investigation for the treatment of hematologic malignancies such as AML, MDS, primary central nervous system lymphoma (PCNSL), and other non-Hodgkin lymphomas. Emavusertib is also being studied in a range of solid tumor malignancies including pancreatic ductal adenocarcinoma (PDAC), colorectal cancer (CRC), and melanoma brain metastases (MBM). At the 3rd Annual IRAK4 in Cancer Symposium, held virtually on September 26, 2024, clinicians and researchers gathered to discuss the oncologic role of IRAK4 and the emerging clinical profile of IRAK4 inhibition.

## Highlights From the Symposium

### IRAK4 in Myeloid Malignancies

B-cell lymphoma 2 inhibitors (BCL2i), such as venetoclax, are highly active in AML, with particular utility in older patients who may be unfit for intensive chemotherapy.^23–25^ However, patients often develop resistance to venetoclax,^25^ and research is ongoing to determine these bypass mechanisms and evaluate combination regimens to prevent or overcome BCL2i resistance. Dr. Marina Konopleva discussed the mechanisms underlying BCL2i resistance in myeloid malignancies and the potential for emavusertib to synergize with venetoclax and overcome venetoclax resistance. One proposed mechanism of BCL2i resistance is activation of intracellular signaling pathways through mutations in *FLT3* or in the RAS/MAPK pathway. Another is increased expression/stabilization of other members of the antiapoptotic BCL2 family, myeloid cell leukemia-1 (MCL-1) and B-cell lymphoma-extra large (BCL-xL).^25^

Dr. Konopleva described preclinical evidence identifying potential approaches to overcome venetoclax resistance. Inhibition of MCL-1 and CLKs/DYRKs has been shown to restore venetoclax sensitivity in venetoclax-resistant AML cell lines and patient-derived xenografts, respectively.^21,26^ Inhibition of FLT3 signaling reduces BCL-xL and MCL-1 expression via the PI3K/AKT, RAS/MAPK, and STAT5 pathways and sensitizes *FLT3*-mutated cells to veneto-clax.^25,27^ Dr. Konopleva further presented data showing that combinations of emavusertib, azacitidine, and venetoclax target multiple potential resistance mechanisms in preclinical models; addition of emavusertib potentiated the antitumor effects of azacitidine and venetoclax in azacitidine- and venetoclax-resistant AML cell lines.^28^ These preclinical data support the potential for emavusertib in patients with resistance to venetoclax and azacitidine.

Dr. Guillermo Garcia-Manero reviewed the MDS treatment landscape, noting a need for novel options and combinations.^29^ Dr. Garcia-Manero presented emavusertib clinical outcomes from the ongoing Phase 1/2 dose-escalation and -expansion TakeAim Leukemia study (NCT04278768), which is evaluating the safety and efficacy of emavusertib in patients with relapsed/refractory (R/R) AML or R/R high-risk MDS (NCT04278768, Table 1).^30^ Since the symposium, an updated dataset was presented at the 66th American Society of Hematology (ASH) Annual Meeting, described in the following section.

Dr. Eric S. Winer reviewed the current state of AML and the treatment landscape, where the introduction of novel therapies has led to incremental improvements, but there remains substantial opportunity to improve patient outcomes by deepening responses, reducing relapses, and establishing more efficacious treatment regimens for patients unable to tolerate intensive chemotherapy.^23, 31–34^ Inhibition of FLT3 is a mainstay in AML, both because it is the most commonly mutated gene in primary AML and because *FLT3* mutations ( *FLT3*m) drive common resistance pathways.^2^ The combined inhibition of IRAK4, FLT3, CLKs, and DYRK1A makes emavusertib a promising potential intervention in AML due to its potential to affect multiple AML-related pathways. Dr. Winer presented data from patients with AML in the TakeAim Leukemia study; updated data have since been presented at the 66th ASH Annual Meeting, described in the following section.

In frontline AML, the combination of venetoclax and azacitidine has become a common treatment approach, especially for older patients. However, patients often progress, and regimens promoting deeper responses are needed.^23,24^ Dr. Klaus Metzeler described the ongoing CA-4948-104 trial, which is a Phase 1, single-arm study assessing the safety, tolerability, and efficacy of emavusertib plus venetoclax plus azacitidine in patients ≥ 60 years of age with AML who achieved minimal residual disease (MRD)-positive complete response (CR) or CR with partial hematologic recovery (CRh) with venetoclax plus azacitidine frontline therapy (EUCTR#2023-505828-58).^35^ In patients who have achieved a CR, better outcomes are associated with those who were MRD-negative (MRD of < 10^−3^ or < 1 residual blast per 1,000 leukocytes) than those who were MRD-positive.^24^ The addition of emavusertib to venetoclax and azacitidine regimens has the potential to increase achievement of MRD-negativity, supported by preclinical evidence of emavusertib synergy with both agents.^28^

### TakeAim Leukemia Trial Outcomes Presented Since the Symposium

**Figure 2:**
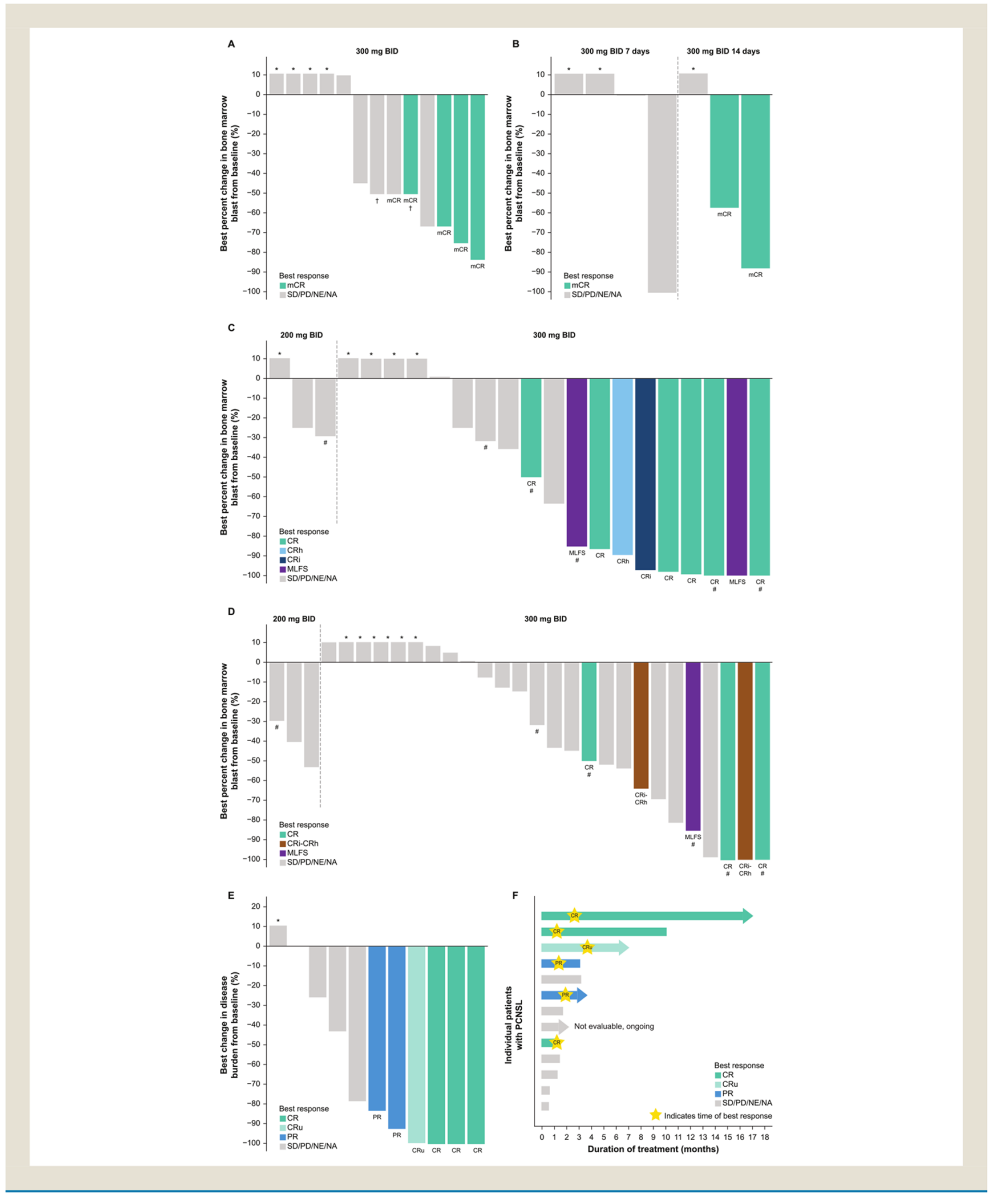
Antitumor activity of emavusertib in hematologic malignancies. Recently presented data of best change in tumor burden in patients who had 1-2 prior lines of therapy and received emavusertib monotherapy with (A) high-risk MDS with SFm who received continuous dosing of emavusertib 300 mg BID,^36^ (B) high-risk MDS who received a reduced schedule regimen of emavusertib 300 mg BID,^36^ (C) AML with *FLT3m* who received continuous dosing of emavusertib 200 or 300 mg BID, ^37^ and (D) AML with SFm who received continuous dosing of emavusertib 200 or 300 mg BID.^37^ (E) Best change in tumor burden and (F) duration of treatment for patients with PCNSL who had previously received BTKi therapy, who received emavusertib 100-300 mg BID plus ibrutinib. *Indicates the best percentage change from baseline > 10%. ^#^Indicates patients having both SFm and *FLT3m*. ^†^Indicates patients who had previously received venetoclax. AML = acute myeloid leukemia; BID = twice daily; BTKi = Bruton’s tyrosine kinase inhibitor; CR = complete response; CRh = CR with partial hematologic recovery; CRi = CR with incomplete hematologic recovery; CRu = CR unconfirmed; *FLT3m* = FMS-like tyrosine kinase 3 mutation; mCR = marrow CR; MDS = myelodysplastic syndrome;MLFS = morphologic leukemia-free state; NA = not available; NE = not estimable; PCNSL = primary central nervous system lymphoma; PD = progressive disease; PR = partial response; SD = stable disease; SFm = spliceosome factor mutations.

Updated data from the TakeAim Leukemia trial were presented at the 66th ASH Annual Meeting. As of an October 12, 2024 data cutoff, a total of 151 patients had received 200-500 mg twice daily (BID) emavusertib monotherapy. Grade ≥ 3 treatment-related adverse events (TRAEs) were reported in 50 patients (33%) across the population, and in 36 of 99 patients (36%) who had received the recommended Phase 2 dose of 300 mg BID. Of the 49 patients with high-risk MDS, 14 had spliceosome factor mutations (SFm), had received 1-2 prior lines of therapy, and received emavusertib 300 mg BID. In this 14-patient cohort, 5 patients (36%) achieved a marrow CR (mCR) (Figure 2).^16^

A newly opened Phase 2a dose-expansion higher-risk MDS cohort in the TakeAim Leukemia trial is assessing the safety, tolerability, effect on bone marrow blast count, and hematologic parameters of emavusertib 300 mg BID dosed for either 7 or 14 days of a 28-day cycle. As of the October 12, 2024 data cutoff, 4 patients in the 7-day/cycle dose group and 3 patients in the 14-day/cycle dose group had been treated, respectively. Two of 3 patients (67%) in the 14-day dose group achieved mCR.^36^

Data presented from TakeAim Leukemia included those from 48 patients with R/R AML with *FLT3m* ( *n* = 21) and/or *U2AF1* or *SF3B1* SFm ( *n* = 31) who had received 1-2 lines of prior therapy and received emavusertib 300 mg BID (data cutoff: October 31, 2024). Five patients had both *FLT3m* and SFm and are included in both populations. A composite CR (CR + CRh + CR with incomplete hematologic recovery [CRi]) was achieved by 8 of 21 patients (38%) with *FLT3m* AML. The overall response rate (ORR, CR + CRh + CRi + morphologic leukemia-free state [MLFS]) was 48% (10/21 patients; Figure 2). Of these 10 responders, prior treatments included BCL2i in 5 patients (50%), FLT3 inhibitors in 6 patients (60%), and hypomethylating agents (HMA) in 6 patients (60%).^37^ In patients with SFm, the composite CR rate was 16% (5/31 patients), and the ORR was 19% (6/31 patients). Of the 6 responders, 4 (67%) received prior BCL2i therapy, and 5 (83%) received prior HMA therapy. Four of the 5 patients with both SFm and *FLT3m* responded (3 CR, 1 MLFS).^37^ Additional molecular analyses (data cutoff: February 26, 2024) have shown disease-modifying activity with emavusertib treatment, including a reduction in frequency or elimination below the limit of detection of the oncogenic FLT3-internal tandem duplication allele,^38^ which is associated with a worse prognosis.^19^

The safety profile of emavusertib in the TakeAim Leukemia trial (data cutoff: October 31, 2024) was manageable. Grade ≥3 TRAEs were reported in 34 of the total 102 patients with AML who received 200-500 mg BID (33%) and in 30 of 92 (33%) patients who received 200-300 mg BID emavusertib; Grade ≥3 TRAEs were primarily hematologic in nature. The most common Grade ≥3 TRAEs were elevated blood creatine phosphokinase (6 patients; 5.9%), neutropenia (6 patients; 5.9%), and anemia (5 patients; 4.9%). There was a single case of laboratory-defined clinical rhabdomyolysis and 2 investigator-reported events of rhabdomyolysis that did not meet laboratory-defined criteria.^37^

### IRAK4 in Lymphoid Malignancies

Dr. Han Tun presented preclinical and clinical data that support targeting IRAK4 in PCNSL. TLRs and B-cell receptors (BCRs) are critical for B-lymphocyte specification, and involved in transformation of malignant B-lymphocytes.^39^ Approximately 80% of PCNSL and 29% of systemic activated B-cell–like diffuse large B-cell lymphoma (DLBCL) harbor *MYD88*-L265P mutations, which cause constitutive activation of MYD88 and thus the myddosome (including IRAK4) and downstream NF- *κ*B activation.^17^ The critical role of IRAK4 within the myddosome creates an attractive target for IRAK4 inhibitors by potentially disrupting aberrant signaling from activated MYD88. Inhibition of the MYD88/myddosome complex via emavusertib in combination with the inhibition of BCR signaling with ibrutinib has the potential for synergistic effects in PCNSL. Preclinical studies in mouse PCNSL models show that emavusertib reaches therapeutically relevant dose levels in cerebrospinal fluid and tumor-bearing brain parenchyma and has single-agent activity; emavusertib has shown synergistic activity in combination with ibrutinib in MYD88-mutated lymphoma cell lines and with either acalabrutinib or zanubrutinib in an activated B-cell (ABC) DLBCL cell line.^17,40,41^ Antilymphoma activity has been seen in both *MYD88*-L265P and *MYD88* wildtype lymphoma models.^16,40,42^

Dr. Tun presented new data from the July 10, 2024 data cutoff from the ongoing study of emavusertib plus ibrutinib in patients with R/R PCNSL (TakeAim Lymphoma; NCT03328078). As of the data cutoff, 13 patients who had received prior Bruton’s tyrosine kinase inhibitor (BTKi) therapy were treated. Grade ≥ 3 TEAEs were reported in 4 patients (30.8%). Treatment was ongoing for 1 patient who had not yet had a response assessment and thus was considered not evaluable for response. Across the 12 response-evaluable patients, 4 achieved a CR, 1 of which had not yet been confirmed by a second postbaseline assessment, and 2 achieved partial responses (ORR of 50%). A duration of response (DOR) of 9 months was reported in 1 patient, and an ongoing DOR of *>* 12 months was reported in another. For the combination of emavusertib plus BTKi, Dr. Tun noted that future areas of investigation could include: (1) concurrent therapy in R/R PCNSL or newly diagnosed PCNSL, (2) maintenance therapy for high-risk patients after induction therapy, autologous stem cell therapy, or chimeric antigen receptor-T maintenance, (3) treatment for elderly patients or those that may not tolerate intensive chemotherapy, and (4) part of a combination with other therapeutic agents such as immunomodulatory drugs and chemotherapy.

Beyond PCNSL, lymphomas such as ABC DLBCL and Walden-ström macroglobulinemia also feature high rates of *MYD88*-L265P mutation.^10^ The response duration with ibrutinib monotherapy in R/R ABC DLBCL is ephemeral (median progression-free survival of 1.64 months);^43^ co-inhibition of TLR and BCR in these patients could result in more durable responses. Encouraging early outcomes for emavusertib and ibrutinib combination regimens in PCNSL support further investigation of this combination more broadly in *MYD88-*mutant B-cell lymphomas.

### IRAK4 in Solid Tumors

Dr. Kian-Huat Lim discussed the potential of IRAK4 as a therapeutic target in gastrointestinal malignancies, specifically PDAC and CRC. Almost all patients with PDAC are treated with conventional therapies with high toxicity and without curative intent.^44^ Immunotherapy has not generally been an effective approach to treat PDAC, partly due to the activity of NF- *κ* B, transforming growth factor- *β*, and other pathways in PDAC cells leading to the formation of a fibrotic stoma that shields the tumor and establishes a protective tumor microenvironment.^44^ Activated IRAK4 is associated with poorer prognosis in PDAC and gastrointestinal cancers; IRAK4 drives multiple pathogenic processes in PDAC, including NF-*κ*B activation, epithelial-mesenchymal transition, and chemoresistance.^45–47^ Additionally, knockout of IRAK4 in preclinical PDAC models resulted in alterations to the tumor immune microenvironment, including reduced tumor-associated macrophages and reduced cancer-associated fibroblasts. Furthermore, in mouse models of PDAC and CRC, addition of IRAK4 inhibition to chemotherapy (gemcitabine plus paclitaxel for PDAC, fluorouracil plus oxaliplatin for CRC) and/or immunotherapy (anticytotoxic T-lymphocyte associated protein 4 plus gemcitabine with or without antiprogrammed cell death protein 1 [PD-1]) resulted in improved tumor responses, supporting the clinical investigation of emavusertib-containing combination regimens for these malignancies.^45,46^

Combinations of emavusertib with other anticancer agents in gastrointestinal malignancies are currently being evaluated in several investigator-sponsored clinical trials. Dr. Lim described ongoing clinical trials of emavusertib combined with chemotherapy, immunotherapy, and/or angiogenesis inhibitor regimens in patients with CRC (emavusertib plus FOLFOX [folinic acid, 5-fluorouracil, oxaliplatin chemotherapy] plus bevacizumab; NCI ETCTN 10655, NCT06696768), PDAC (emavusertib plus gemcitabine plus nab-paclitaxel; NCI ETCTN 10522, NCT05685602), and gastric or esophageal cancer (emavusertib plus anti-PD–1 [nivolumab] plus modified FOLFOX7 with or without trastuzumab; NCT05187182).

Dr. Bently Doonan discussed compelling preclinical evidence for combining inhibition of IRAK4 and PD-1 in the treatment of multiple MBM. Metastatic melanoma is associated with a high risk of the development of brain metastases.^48–50^ The MBM tumor microenvironment is highly proinflammatory and commonly features TLR pathway activation.^42^ Consistent with the role for TLR signaling in MBM, MYD88 promotes tumor resistance to immune checkpoint inhibitors in melanoma, supporting the rationale for IRAK4 inhibition as a novel therapeutic option to disrupt the inflammatory cascade, including in combination with immune checkpoint inhibitors.^17^

As monotherapy, emavusertib demonstrated modest activity in murine models of aggressive MBM,^17^ and emavusertib/immune checkpoint inhibitor combination regimens are being studied. Dr. Doonan presented novel research in a murine melanoma model that demonstrated a synergistic effect of combining emavusertib and pembrolizumab, including increased MBM tumor-infiltrating lymphocytes, interferon gamma expression, and overall survival (article in press). Reduction in inflammation and activation of the immune system resulted in immune-mediated tumor destruction. These promising preclinical findings motivated a Phase 1/2 study of emavusertib plus pembrolizumab following stereotactic radiosurgery in patients with MBM (NCT05669352). This trial is active and currently enrolling.

### Current and Future Opportunities for IRAK4 Inhibition

In roundtable discussions during the meeting, participating experts discussed current and future opportunities for IRAK4 inhibition. Speakers agreed that, based on initial clinical outcomes for emavusertib, IRAK4 inhibition continues to be well tolerated in the clinical setting and shows robust activity in R/R hematologic malignancies AML, MDS, and PCNSL. Speakers were also encouraged by the preclinical and emerging clinical data for IRAK4 inhibition in solid tumors (Table 1). Beyond current clinical efforts, speakers highlighted the rationale for the investigation of emavusertib and other IRAK4 inhibitors in the frontline setting in AML, MDS, and PCNSL, especially in patients who are not eligible for high-intensity therapies.

There is a need for novel agents that directly target the signaling involved in the pathogenesis of both primary disease and resistance mechanisms. As discussed throughout the symposium, the frequency of IRAK4-dependent NF- *κ* B activation in cancers makes IRAK4 a key target for novel anticancer therapies. Even in malignancies that often respond to current treatment regimens, IRAK4 inhibition has the potential to improve response rates and increase the durability of responses through bypassing current resistance mechanisms.

Emerging evidence indicates that IRAK4 inhibition can both resensitize cancers to and synergize with current standards of care due to its immunomodulatory effects and targeting of common resistance mechanisms. Speakers were encouraged by the potential of IRAK4 inhibition, as shown by emavusertib, to sensitize various chemorefractory tumors such as PCNSL, PDAC, and MBM to other therapeutic agents. IRAK4 inhibition combined with other agents is currently being assessed in multiple ongoing clinical trials. Across treatment areas, speakers saw the potential of IRAK4 inhibitor-containing doublet and triplet regimens to achieve long- lasting remission. For example, an ongoing trial assessing the triplet regimen of emavusertib plus venetoclax and azacitidine will specifically assess if IRAK4 inhibition can support achievement of MRD negativity and eligibility for autologous stem cell transplant for applicable malignancies. Outcomes from this trial may motivate further investigation of the combination in patients with AML or AML/MDS who are ineligible for intensive chemotherapy.

The safety profile of IRAK4 inhibition in cancer is also emerging, predominantly based on clinical outcomes for emavusertib. To date, treatment with emavusertib continues to be tolerable. The potential for myelosuppression with regimens that include FLT3 inhibition was specifically discussed, as FLT3 is an important target in hematologic malignancies but also plays a role in myeloid differentiation. The degree to which myelosuppression associated with emavusertib is due to its activity against IRAK4 or FLT3 remains to be determined. Speakers discussed the benefits of alternative dose schedules and dose reductions for therapies used in combination regimens as approaches to mitigate toxicities. Overall, speakers found the adverse events associated with emavusertib to be manageable.

Clinical outcomes for emavusertib to date suggest that IRAK4 inhibitor monotherapy may be particularly promising in patients with hematologic malignancies harboring targeted *FLT3* or spliceo-some mutations. The activity of emavusertib to inhibit IRAK4, FLT3, CLK, and DYRK1A may also provide benefit in specific genetic subsets of patients with hematologic malignancies.

The 3rd Annual IRAK4 in Cancer Symposium brought further understanding to the biologic role of IRAK4 in many malignancies, while highlighting current clinical data of the IRAK4 inhibitor emavusertib. Ongoing and future research and clinical trials will further elucidate the therapeutic potential of IRAK4 inhibition in cancer.

## Figures and Tables

**Table 1:** Select Ongoing Clinical Trials of IRAK4 Inhibitors

Trial Name and NCT Number	Phase/Design	Patient Population	Planned Enrollment	Primary Endpoints
TakeAim Leukemia Study, CA-4948-102 NCT04278768	Phase 1/2a dose-escalation and -expansion: Emavusertib monotherapy	Relapsed/refractory AML/high-risk MDS including patients with specific mutations in *FLT3* or the spliceosome	366	• Phase 1: MTD, RP2D • Phase 1b: safety • Phase 2a: anticancer activity
CA-4948-104 EU CT: 2023-505828-58-00	Phase 1 open-label study: Emavusertib as add-on therapy for existing first-line azacitidine + venetoclax	AML following achievement of CR or CRh with MRD positivity	9-18	• Safety
R289 in R/R LR-MDS NCT05308264	Phase 1/2 dose-escalation study of R289	Relapsed/refractory LR MDS	86	• Safety and tolerability
TakeAim Lymphoma Study, CA-4948-101 NCT03328078	Phase 1/2 dose-escalation and -expansion • Part A1: escalating doses of emavusertib monotherapy • Part A2: escalating doses of emavusertib + ibrutinib • Part B expansion: RP2D of emavusertib plus ibrutinib • Part C: emavusertib 200 mg BID, ibrutinib 560 mg QD, or emavusertib 200 mg BID plus ibrutinib 560 mg QD	Part A1 and A2: R/R non-Hodgkin lymphoma Part B: R/R PCNSL Part C: R/R PCNSL	152	• Part A: safety and tolerability of emavusertib ± ibrutinib; MTD and RP2D of emavusertib ± ibrutinib • Part B: ORR
NCI ETCTN 10522, NCT05685602	Phase 1 dose-escalation and -expansion: Emavusertib + gemcitabine/nab-paclitaxel	Metastatic or unresectable PDAC	36	• Safety (DLT rate)
NCI ETCTN 10655, NCT06696768	Phase 1 dose-escalation and -expansion: Emavusertib + mFOLFOX6 + bevacizumab	1L metastatic colorectal cancer	24	• Safety (DLTs, TEAEs)
NCT05187182	Phase 1 dose-escalation and -expansion: Emavusertib + PD-1 inhibitor (nivolumab or pembrolizumab) + mFOLFOX7 ± trastuzumab	Unresectable gastric and esophageal cancer	42	• Safety • Expansion dose emavusertib in combination regimen
NCT05669352	Phase 1/2: Emavusertib + pembrolizumab following SRS	MBM (high or low burden disease)	29	• Reduction in need for repeated intercranial intervention 1 year after initial SRS

Information in this table is from Clinicaltrials.gov as of May 8, 2025, or from the IRAK4 in Cancer Symposium.

Abbreviations: 1L = first-line; AML = acute myeloid leukemia; CR = complete response; CRh = complete response with partial hematologic recovery; DLT = dose limiting toxicity; IRAK4 = interleukin-1 receptor-associated kinase 4; LR = low risk; MBM = melanoma brain metastases; MDS = myelodysplastic syndrome; mFOLFOX = modified folinic acid, 5-fluorouracil, oxaliplatin chemotherapy; MTD = maximum tolerated dose; NCI ETCTN = National Cancer Institute Experimental Therapeutics Clinical Trials Network; ORR = objective response rate; PCNSL = primary central nervous system lymphoma; PD-1 = programmed cell death protein 1; PDAC = pancreatic ductal adenocarcinoma; R/R = relapsed/refractory; RP2D = recommended Phase 2 dose; QD = once daily; SRS = stereotactic radiosurgery; TEAE = treatment-emergent adverse event.
